# Nystatin-like *Pseudonocardia* polyene B1, a novel disaccharide-containing antifungal heptaene antibiotic

**DOI:** 10.1038/s41598-018-31801-y

**Published:** 2018-09-11

**Authors:** Hye-Jin Kim, Chi-Young Han, Ji-Seon Park, Sang-Hun Oh, Seung-Hoon Kang, Si-Sun Choi, Jung-Min Kim, Jin-Hwan Kwak, Eung-Soo Kim

**Affiliations:** 1 Department of Biological Engineering, Inha University, Incheon, 22212 Korea; 2 Jeil Pharmaceutical Co., Ltd., Yongin-si, Gyeonggi-do, 17172 Korea; 3 School of Life Science, Handong Global University, Pohang, 37554 Korea

## Abstract

Polyene macrolides such as nystatin A1 and amphotericin B belong to a large family of very valuable antifungal polyketide compounds typically produced by soil actinomycetes. Recently, nystatin-like *Pseudonocardia* polyene (NPP) A1 has been identified as a unique disaccharide-containing tetraene antifungal macrolide produced by *Pseudonocardia autotrophica*. Despite its significantly increased water solubility and decreased hemolytic activity, its antifungal activity remains limited compared with that of nystatin A1. In this study, we developed NPP B1, a novel NPP A1 derivative harboring a heptaene core structure, by introducing two amino acid substitutions in the putative NADPH-binding motif of the enoyl reductase domain in module 5 of the NPP A1 polyketide synthase NppC. The low level NPP B1 production yield was successfully improved by eliminating the native plasmid encoding a polyketide biosynthetic gene cluster present in *P*. *autotrophica*. *In vitro* and *in vivo* antifungal activity and toxicity studies indicated that NPP B1 exhibited comparable antifungal activity against *Candida albicans* and was less toxic than the most potent heptaene antifungal, amphotericin B. Moreover, NPP B1 showed improved pharmacokinetic parameters compared to those of amphotericin B, suggesting that NPP B1 could be a promising candidate for development into a pharmacokinetically improved and less-toxic polyene antifungal antibiotic.

## Introduction

Polyenes typically comprise a polyketide core macrolactone ring with 20–40 carbon atoms including 3–8 conjugated double bonds. The antifungal drugs primarily used to treat systemic fungal infections are polyene macrolides such as the tetraene-containing nystatin A1 and heptaene-containing amphotericin B^1,2^. The primary antifungal mechanism of polyene antimicrobials is believed to be dependent on the interactions between antibiotic molecules and ergosterol that appear to occur through the polyene region of the macrolactone core^3,4^. The relatively high toxicity of polyene antimicrobials toward mammalian cells, probably due to cholesterol-binding capacity and the poor distribution of these molecules in tissues, has limited their use for antifungal therapy.

Amphotericin B is regarded as the last line antifungal drug for the treatment of invasive fungal infections^5^, ever since systemic antifungal drugs were first successfully used clinically in the 1950s following the approval of amphotericin B deoxycholate^6^. Although resistance is rarely acquired against amphotericin B, its extreme toxicity limits its broad use^7,8^. Considerable efforts have been made to develop less toxic amphotericin B derivatives using chemical methods^9–11^. Nonetheless, attempts to rationally predict and design a less-toxic polyene structure that preferentially binds to ergosterol and not cholesterol have not been achieved.

NPP A1, produced by a rare actinomycetes, *Pseudonocardia autotrophica*, showed 300-fold higher water-solubility and 10-fold reduced hemolytic activity than those of the structurally similar nystatin A1 but exhibited approximately 50% lower antifungal activity^12^. In contrast to the heptaene-containing amphotericin B, the macrolide core structures of both NPP A1 and nystatin A1 are tetraenes, which have a saturated C28-C-29 bond in the polyene region. The number of conjugated double bonds has been presumed to account for the higher fungicidal activity and the broad spectrum antifungal effects^13,14^. The data obtained for S44HP, the heptaene of nystatin A1, support that modification of the polyene region increases not only the antifungal activity but also toxicity^13,15^.

In this study, we successfully developed the heptaene form of NPP A1 named NPP B1 by manipulating the specific polyketide enoyl reductase (ER) domain in the NPP A1 biosynthetic pathway gene, followed by substantial efforts to enhance the NPP B1 production yield. The *in viv*o efficacy against *C*. *albicans*, as well as the *in vivo* toxicity and pharmacokinetics were examined. We expect this study to be the basis for the development of a novel less-toxic disaccharide heptaene macrolide of *P*. *autotrophica*, which has comparable antifungal efficacy to that of the most potent heptaene antifungal antibiotic, amphotericin B.

## Results

### NPP B1 biosynthesis by engineering polyketide synthase (PKS) ER domain in module 5 (ER5)

**Figure 1:**
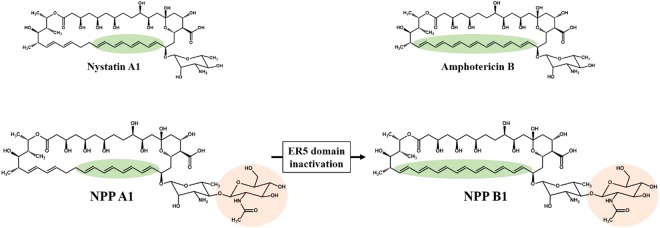
Polyene macrolides investigated in this study. Nystatin A1, a typical tetraene polyene macrolide produced by *Streptomyces noursei*; amphotericin B, the most potent polyene macrolide produced by *Streptomyces nodosus*; nystatin-like *Pseudonocardia* polyene **(**NPP) A1, produced by *Pseudonocardia autotrophica* wild-type; NPP B1, produced by enoyl reductase (ER) domain in module 5 (ER5) inactivation mutant of *P*. *autotrophica*.

During biosynthesis of the NPP A1 polyketide backbone, the ER domain in module 5 (ER5) of NppC is responsible for the nicotinamide adenine dinucleotide phosphate (NADPH)-mediated reduction of the C28-C29 unsaturated bond. NPP B1, a heptaene form of NPP A1, was previously developed by the in-frame deletion of the ER5 domain in *P*. *autotrophica*^12^. High-performance-liquid chromatography (HPLC) and electrospray ionization-mass spectrometry (ESI-MS) analysis of NPP B1 indicated a heptaene structure containing a di-sugar moiety. Unfortunately, however, the production yield of NPP B1 (0.34 mg/L, 5-day flask cultivation) was extremely poor compared with that of the wild-type (8.91 mg/L, 5-day flask cultivation) (Fig. 1), implying that the in-frame deletion of the ER5 domain might affect the three-dimensional (3D) structure of NPP polyketide synthase, rendering it less efficient in subsequent biosynthetic steps.

**Figure 2:**
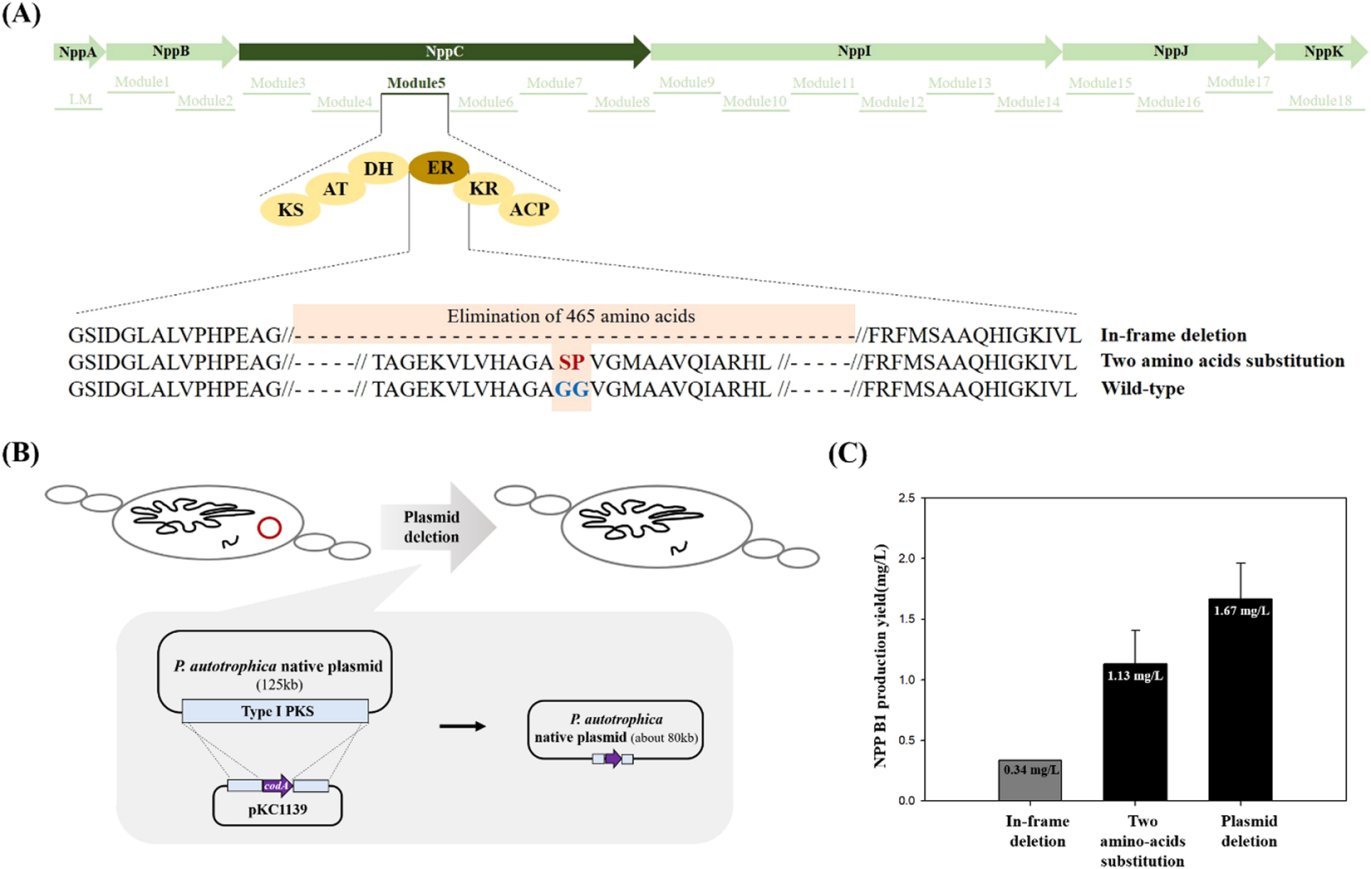
Development of nystatin-like *Pseudonocardia* polyene (NPP) B1 overproducing strains in *Pseudonocardia autotrophica*. (**A**) Inactivation scheme of enoyl reductase (ER) domain in module 5 (ER5) of *nppC* gene; KS, ketosynthase; AT, acyltransferase; DH, dehydratase; ER, enoyl reductase; KR, ketoreductase; ACP, acyl carrier protein. (**B**) Deletion scheme of *P*. *autotrophica* originated plasmid. (**C**) Comparison of NPP B1 production yields with newly constructed NPP B1 overproducing strains.

This low titer problem was overcome by generating an alternative NPP B1 producing strain by substituting only two amino acids in the NADPH-binding motif in the ER5 domain of NppC. The site-specific GG5036SP mutant of NppC was generated in *P*. *autotrophica*, similar to that used in the GG5073SP mutant in *Streptomyces noursei*, which exclusively produced a heptaene version of nystatin A1, S44HP in *S*. *noursei*^14^. The GG5036SP mutant of NppC was constructed by homologous recombination based on the temperature-sensitive plasmid, pKC1139 (Fig. 2A). The same substitution of amino acids in the ER5 domain using the pKC1132, which did not require heat-mediated recombination, was also attempted to eliminate heat-mediated spontaneous mutations (Fig. S1). These site-specific NPP ER5 mutants generated by both pKC1132 and pKC1139 showed an improved NPP B1 production yield by approximately 3.2-fold compared with that obtained by the previously constructed in-frame deletion of ER5 domain mutant (Fig. 2C). These results indicate that the site-specific substitution of amino acids in the NADPH-binding motif in the ER5 domain is a more effective strategy for the development of NPP B1 producing strain than those previously attempted, and the type of plasmids for recombination do not affect NPP B1 production.

### Improvement of NPP B1 production yield

Although the NPP B1 producing strain was developed by modifying amino acids in the ER5 domain, NPP B1 production yield in this mutant was still <10% of the NPP A1 production yield of the *P*. *autotrophica* wild-type (Fig. S2A). The quantitative reverse transcription-polymerase chain reaction (qRT-PCR) analysis revealed that the transcriptional repression of PKS genes in the NPP B1-producing strain reduced the NPP B1 production yield compared with that of the wild-type, whereas the transcripts of the NPP pathway-specific regulatory genes (*nppRI*, *nppRII*, *nppRIII*, *nppRIV*, *nppRV*, and *nppRVI*) were not significantly changed in both the wild-type and NPP B1-producing strain (Fig. S2B). To improve the NPP B1 production yield, several strategies including overexpression of the pathway-specific regulatory gene (*nppRIV*, Fig. S3), deletion of global antibiotic downregulator, WblA-ortholog (Fig. S4), *in situ* screening of NPP B1 random mutant colonies using Raman microspectroscopy^16^ (Fig. S5 and Table S1), co-culture with *Corynebacterium glutamicum*, and cultivation with xenobiotics such as triclosan, were attempted (Fig. S6). Despite various efforts, no noticeable improvement in the NPP B1 production has been achieved.

Meanwhile, two native plasmids (circular and linear forms of approximately 125 kb and 8 kb, respectively) were identified using whole genome sequencing of *P*. *autotrophica*. Based on the antibiotics and secondary metabolite analysis shell (antiSMASH) database, it was predicted to be a novel type I polyketide biosynthetic pathway displaying approximately 5% similarity to the kedarcidin biosynthetic gene cluster located in a native 125 kb circular plasmid (Fig. S7). This observation implied that the presence of a giant plasmid could be the metabolic burden in the cell, and the plasmid-born type I polyketide biosynthetic pathway might be the competing pathway during the NPP B1 biosynthesis. Therefore, the native 125 kb circular plasmid was eliminated using the homologous recombination method with the negative selection marker, CodA, in the NPP B1-producing *P*. *autotrophica* strain. The result showed that the removal of the native plasmid increased the NPP B1 production by 147% compared with that of the site-specific NPP ER5 mutants, which is 4.9-fold higher than that of the ER5 domain deletion mutant (Fig. 2B,C). These results indicate that deletion of the NPP B1 competing pathway is an effective method for NPP B1 strain improvement in *P*. *autotrophica*.

### *In vitro* antifungal activity and hemolytic toxicity

The *in vitro* antifungal activity of NPP B1 against four different *C*. *albicans* strains was approximately 2- to 4-fold higher than that of NPP A1 and nystatin A1 (Table 1). However, NPP B1 exhibited a 2-fold lower antifungal activity than that of amphotericin B (Table 1). Moreover, the hemolytic activity of NPP B1 was confirmed to be slightly decreased compared with that of amphotericin B (Table 1). Hepatotoxicity, one of the major toxic side effects associated with polyene macrolides, was also evaluated in two human hepatocytes (Hep3B and HepG2 cells), and the results indicated that *in vitro* hepatotoxicity of NPP B1 was not improved comparing with AmB (Fig. S8). These results are consistent with those previously observed for amphotericin B and S44HP, which both possess a heptaene structure^13,15^. The microsomal stability of NPP B1 was investigated using human liver microsome. We observed that after an 80-min incubation, the amount of the remaining NPP B1 was 28.2% and the half-life (T_1/2_) was calculated to be 36.5 min (Table S2).

**Table 1:** *In vitro* antifungal activity and toxicity of polyene macrolides.

	Nystatin A1	NPP A1	Amphotericin B	NPP B1
**Antifungal activity (MIC**, **µg/mL)**^a^				
*Candida albicans* KCTC7965	4	16	1	2
*C*. *albicans* SC5314	4	16	1	4
*C*. *albicans* SL28	4	16	1	4
*C*. *albicans* SL38	4	16	1	2
*Cryptococcus humicola* ATCC9949	—	—	0.5	1
*Saccharomyces cerevisiae* ATCC9035	—	—	1	2
**Hemolytic activity (MHC**, **µg/mL)**^**b**^				
	66.17 ± 0.90	459.35 ± 32.5	4.65 ± 0.17	13.60 ± 0.19

^a^MIC, minimum inhibitory concentration (values resulting in no visible growth of *C*. *albicans*).

^b^MHC, minimum hemolytic concentration (values causing 90% hemolysis against horse blood cells ± percentage standard deviation).

### *In vivo* efficacy, pharmacokinetics, and toxicity

**Figure 3:**
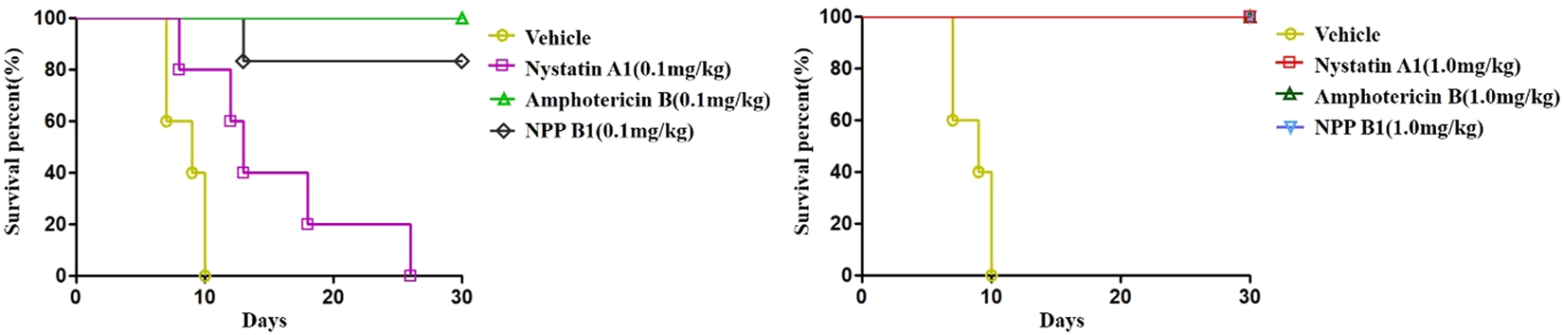
*In vivo* efficacy of polyene macrolides. The survival rates of mice infected with *Candida albicans* after treatment with low and high concentrations (left and right, respectively) of polyene macrolides.

To evaluate the potential of NPP B1 as a drug candidate, we decided to further evaluate its *in vivo* efficacy and toxicity. We screened for the optimal pathogenic *C*. *albicans* strain and performed a minimal lethal dose (MLD) test to establish an appropriate candidiasis-infected mouse model. *C*. *albicans* SC5314 showed the highest virulence among two standard strains and three clinical strains via tail vein injections to 3-week-old male ICR mice. Furthermore, the optimal concentration for infection of *C*. *albicans* was determined to be 1 × 10^6^ CFU/mouse based on the survival rate of ICR mice, which was approximately 2 weeks after infection (Fig. S9). Our result revealed that the groups treated with low and high NPP B1 doses (0.1 and 1 mg/kg, respectively) showed similar survival rates compared with that of amphotericin B-treated mice, whereas the survival rate gradually decreased in the group treated with low dose (0.1 mg/kg) nystatin A1 (Fig. 3). Therefore, NPP B1 was identified to have a significantly improved antifungal activity compared with that of NPP A1, and comparable to that of amphotericin B. We further characterized the pharmacokinetics of NPP B1 in the SD rats (Table S3) and mice (Table S4). The rat pharmacokinetic profiles of nystatin A1, amphotericin B, NPP A1, and NPP B1 were examined by intravascular administration which were not detected in the plasma following oral administration. The 1 mg/kg dose was studied with nystatin A1, NPP A1 and NPP B1 except for amphotericin B which killed all the tested rats at same dose. After intravenous administration of NPP B1 1 mg/kg to rats, the area under the curve (AUC) was markedly higher than nystatin A1 and the original compound, NPP A1, and the elimination half-life (T_1/2_) was 14.5 hr longer for the NPP B1 than for other compounds (Table S3). Similar pharmacokinetic profiles of nystatin A1, amphotericin B, NPP A1, and NPP B1 were also observed in mice (Table S4). These results imply that NPP B1 is a promising candidate for development as a novel antifungal drug with superior efficacy and improved pharmacological profile.

The acute toxicity of NPP B1 in mice was investigated following a single intravenous administration by monitoring for lethality (Tables 2 and S5). A preliminary toxicity test was conducted to determine the optimal concentration of NPP B1 for toxicity testing. Notably, the median lethal dose (LD_50_) of NPP B1 in both female and male group were calculated to be 2.95 and 2.95 mg/kg, whereas those of amphotericin B were calculated 1.6 and 2.95 mg/kg, respectively (Tables 2 and S5). NPP B1 was also evaluated in a 7-day repeated toxicity study, and demonstrated a higher maximum tolerated dose (MTD) compared with amphotericin B (Tables 2 and S6), thereby suggesting that NPP B1 harboring the unique di-sugar moiety has lower *in vivo* toxicity than amphotericin B. These results were consistent with the data for single and repeated dose toxicity tests of nystatin A1 and NPP A1, where the disaccharide of the polyene structure reduced the toxicity.

**Table 2:** *In vivo* toxicity of polyene macrolides in mice.

Compounds	Dose (mg/kg)^a^	
	LD_50_^b^	MTD^c^
NPP B1	2.95	>1.5
Amphotericin B	2.3	1.5
NPP A1	16.13	5–10
Nystatin A1	5.37	2–4

^a^Treated compound concentrations (low, mid, high) for single intravenous administration, nystatin A1(2.5, 5, 10 mg/kg), NPP A1(7.5, 15, 30 mg/kg), amphotericin B and NPP B1(1, 2.5, 5 mg/kg); 7-day repeated intravenous doses, nystatin A1(1, 2, 4 mg/kg), NPP A1(2.5, 5, 10 mg/kg), amphotericin B (0.5, 1.5 mg/kg) and NPP B1(1.5 mg/kg).

^b^LD_50_, median lethal dose (dose causing 50% mortality).

^c^MTD, maximal tolerated dose.

## Discussion

The polyene macrolide family, including nystatin A1 and amphotericin B, is one of the key classes of antifungal drugs. Especially, amphotericin B has for over half a century been considered the last line of treatment for systemic fungal infections, despite its substantial toxicity^4,5^. The C28-C29 unsaturated bond in amphotericin B was presumed to account for the higher fungicidal activity and the broader antifungal spectrum than that of nystatin A1. Therefore, in this study, we generated an NPP B1 strain, which is an improved version of the heptaene-producing NPP A1 mutant, by performing site-specific two-amino acid substitutions in the ER5 domain of *P*. *autotrophica*.

Although only two amino acids at the NADPH-binding site of ER5 were substituted, the PKS gene transcripts responsible for the formation of the macrolactone ring with a range of conjugated double bonds were significantly decreased. This modification reduced the production of NPP B1 by approximately 10-times compared to that of NPP A1 in *P*. *autotrophica* wild-type. Moreover, the transcripts of 128 genes in the ER5 domain mutant strain were increased or decreased with at least 2-fold changes, compared with that of the wild-type (unpublished data). More in-depth comparative transcriptome analyses of the NPP B1 and NPP A1 wild-type strains need to be conducted separately.

The whole genome sequencing and RNA-sequencing analyses indicated that the rare actinomycetes, *P*. *autotrophica*, differs from the typical actinomycetes strain. It was confirmed that the *P*. *autotrophica* genome structure was circular with a 5.8 Mb size, unlike the linear genome of most actinomycetes (8–9 Mb size). Overexpression of the putative PAS-LuxR transcriptional regulator, NppRIV in the *P*. *autotrophica* wild-type and ER5 domain inactivation mutant reduced NPP A1 production or did not significantly change that of NPP B1, respectively. In contrast, previous studies reported that PAS-LuxR transcriptional activators such as AmphRIV and NysRIV shared the same regulatory pattern in *Streptomyces nodosus* and *S*. *noursei*, independently, and boosted the production of polyenes^17,18^. Deletion of the WblA ortholog in the ER5 domain inactivation mutant reduced NPP B1 production. The effect of WblA on secondary metabolite production has been verified in various *Streptomycetes* species^19–22^. The above results also imply that the regulatory system for producing secondary metabolites in *P*. *autotrophica* might be distinct from those of other *Streptomycetes*. The results obtained in coculture with the mycolic acid-containing bacteria, *C*. *glutamicum*, and the efficacy of triclosan, an inhibitor of fatty acid synthesis, were also not very effective in stimulating NPP B1 production^23,24^. Taken together, these results suggest that the fundamental difference and unique characteristics of *P*. *autotrophica* need to be investigated. Fortunately, deletion of the indigenous plasmid (125 kb) encoding an NPP competing for polyketide pathway noticeably enhanced the production yield of NPP B1 in *P*. *autotrophica*.

In this present study, we described for the first time, the *in vitro* and *in vivo* biological activities of NPP B1, the new heptaene version of NPP A1. It is noteworthy that NPP B1 with the unsaturated C28-C29 bond displayed significantly improved *in vitro* and *in vivo* antifungal activity to that of NPP A1. Moreover, NPP B1 showed enhanced pharmacokinetics and reduced toxicity in comparison with the structurally-similar heptaene, amphotericin B. The mechanism of the antifungal activity of polyene macrolides has been proposed to involve simple binding of ergosterol while binding to cholesterol accounts for its toxicity. The preliminary SPR experiments indicate that amphotericin B showed higher affinity for both the ergosterol- and cholesterol-containing membranes than NPP B1, which binds preferentially to ergosterol-containing liposomes^25^ (unpublished data). The cholesterol binding selectivity might translate into differences in the toxicities of amphotericin B and NPP B1, which need to be further verified. In summary, NPP B1 is a promising heptaene alternative to amphotericin B with reduced toxicity, and *in vivo* studies suggest that the addition of extra sugar residue to the heptaene macrolides could improve its pharmacological properties without significantly reducing the antifungal efficacy. This study has made the considerable progress in expanding the structural modification of NPP B1 to improve toxicity as previously described^26,27^, 16-decarboxy-16-methyl NPP B1 and 10-deoxy NPP B1.

## Materials and Methods

### Bacterial strains, plasmids, and growth conditions

The microbial strains and plasmids used in this study are listed in Table S7. *Escherichia coli* strains were cultivated in LB medium supplemented with appropriate antibiotics. Gene deletions and overexpression were performed by conjugations into *P*. *autotrophica* strain as previously reported^12,28^. The *P*. *autotrophica* strains for producing the analogs were cultured using both flask culture and batch fermentation as described elsewhere^27,29^.

### Construction of *P*. *autotrophica* mutant strains

(i) ER5 domain inactivation mutant. The 2.0 kb and 1.9 kb fragments including the NADPH-binding site of the ER5 of NppC were PCR-amplified with the following primers. GG5036SP-1: forward, 5-GAATTCTGCTCTGCACCGGTCGGCTG-3′ and reverse, 5′-GCTAGCACCGGCGTGGACGAGCA-3′ and GG5036SP-2: forward, 5′-GCTAGCCCGGTCGGCATGGCCGCGGTCCA-3′ and reverse, 5′-AAGCTTCCGTGTCCACCGTCATCGCC-3′, introducing *Eco*RI, *Nhe*I, and *Hind*III sites (underlined) into the PCR products. These sites were cloned into pKC1139. This plasmid was used as a template for site-directed mutagenesis using homologous recombination to introduce ER5 domain inactivation. The mutation was first confirmed by restriction analysis (*Nhe*I) of PCR fragments using primers ER5: forward, 5′-CATCGTGCTCGTCGACCT-3′ and reverse, 5′- ACCCAGCTCACCGAGCTC-3′, which were then verified using DNA sequencing.

(ii) Competition pathway deletion. Two 2 kb fragments were PCR-amplified from the *P*. *autotrophica* circular plasmid using primers pPA-1, forward, 5′- CTATGACATGATTACGAATTCTGGTCCACGTCTCTACAACTTCA-3′ and reverse, 5′-AGATGTCAACCTCGAGTGCTGTATCGACAAGGTCAAAGCT-3′; pPA-2, forward, 5′-TCTTGCCTTGCTCGTCGCGATGAGCTGGAAGAATAGGTT-3′ and reverse, 5′-ACGACGGCCAGTGCCAAGCTTAACCTAGGCGAGCACAAGGAC-3′, introducing *Eco*RI and *Hind*III sites (underlined) into the PCR products. These sites were used to clone the PCR fragments into pKC1132. The 1.5 kb fragment was also amplified from pWHU2653^30^ using primers *codA*, forward, 5′-GACGAGCAAGGCAAGACCGAT-3′ and reverse, 5′-CTCGAGGTTGACATCTTTTGCCG-3′ to include the negative selection marker, CodA. Three PCR products were cloned into pKC1132 using the In-Fusion HD cloning kit (Clontech, USA). The resulting plasmid was integrated into the *P*. *autotrophica* ER5 domain inactivation strain and deleted the plasmid by the action of the negative selection marker, CodA.

(iii) WblA ortholog deletion. Two 2 kb fragments were amplified using primers Del_whiB4–1: forward, 5′-GGCAGCGTGAAGCTTCTGCTGGGTCGTCCCGGTC-3′ and reverse, 5′-CTGGTCGCCTCTCGGGCC-3′; Del_whiB4–2: forward, 5′-CCGAGAGGCGACCAGCCCGATAGCGTCAGGCG-3′ and reverse, 5′-CAGGCATGCAAGCTTGACGCGTCGGCGTTCGTAC-3′, introducing the *Hind*III sites (underlined), which were cloned into pWHU2653 using the In-Fusion HD cloning kit. The entire 4 kb insert was excised using *Hind*III and cloned into the pKC1132. The resulting plasmid was introduced, and it induced homologous recombination in the ER5 domain inactivation mutant, and its recombinant strain eliminated the circular plasmid, individually.

### Production, purification, and analysis of polyenes

The polyenes were produced and analyzed using previously described methods^27,29^. The production yield and purity >80% (NPP B1 peak area/areas of all polyene peaks at 405 nm) of the NPP B1 from the recombinant *P*. *autotrophica* strain were determined using HPLC analysis using the amphotericin B standard as a reference. Concentrations of NPP B1 were measured using an API2000 LC-MS/MS system (Applied Biosystems) equipped with TurboIon Spray source operated in the positive ion mode. Sample injection volume was 10 ul and separation was performed on an XTerra MS C18 column (2.1 × 50 mm, 3.5 um; Waters) maintained at 30 °C. The column was developed using the following linear gradient: from 0.1% formic acid/0% acetonitrile/100% deionized (DI) water to 0.1% formic acid/100% acetonitrile/0% DI water. The optimized electrospray ionization parameters were as follow: declustering potential (DP): 151 (NPP B1), 111 (amphotericin B) and 56 V (IS); collision energy (CE): 91 (NPP B1), 83 (amphotericin B) and 29 V (IS); focusing potential (FP): 170 (NPP B1), 280 (amphotericin B) and 360 V (IS); collision cell exit potential (CXP): 2 (NPP B1), 0 (amphotericin B) and 4 V (IS). Nebulizer gas (NEB), curtain gas (CUR) and collision gas (CAD) were set to 45, 10 and 6 psi, respectively. The monitoring ion were set as m/z 1127 → 105 for NPP B1, m/z 924 → 107 for amphotericin B and m/z 237 → 194 for the IS. The scan dwell time was 100 ms for each transition channel. All data were acquired and analyzed using the Analyst software (version 1.5.2, Applied Biosystems).

### *In vitro* antifungal activity, toxicity, and stability test

For the minimum inhibitory concentration (MIC) assay, strains for testing were grown on YPD agar for 24 hours, and subcultured to YPD broth medium. Whole strains were incubated for 18 hours at 35 °C by shaking incubator. Grown organisms were diluted with fresh RPMI 1640 medium to appropriate concentration, 5 × 10^4^ CFU/ml. Cultures were inoculated to 96-well plates containing antifungal medium. All compounds were dissolved using Dimethyl Sulfoxide (DMSO) and diluted broth media. Fungal diluted solution and antifungal medium were mixed at 1:1 ratio so total 200 ul mixed medium was made. Whole experiment plates were incubated for 24 hours at 37 °C. MIC value was determined by the well that showed inhibition of growth completely and revealed the lowest concentration. The MICs were calculated in duplicate using the Clinical and Laboratory Standards Institute M27-A3 microbroth methodology^31^. Hemolysis experiments and the 3-(4,5-dimethylthiazol-2-yl)-5-(3-carboxymethoxyphenyl)-2-(4-sulfophenyl)-2H-tetrazolium (MTS) cell proliferation colorimetric assay were performed following known procedures^12,27,29^.

A metabolic stability experiment was conducted to calculate the rate of disappearance of polyene macrolides following *in vitro* incubation with human microsomes. Working solutions of polyene macrolides were prepared from a 10 mM stock solution in dimethyl sulfoxide (DMSO) diluted to a final concentration of 1 µM in 0.1 M potassium phosphate buffer (pH 7.4). Each incubation mixture included 1 mg/mL human microsomal protein (BD Gentest^TM^, Franklin Lakes, NJ, USA) and 1 mM NADPH. The reactions were terminated at 0, 15, 45, and 80 min. The reaction mixtures were vigorously vortexed and then analyzed using the API2000 LC-MS/MS) system (Applied Biosystems, USA) using an Xterra MS C18 (3.5-µm, 2.1 × 50 mm, Waters).

### Pharmacokinetics, *in vivo* efficacy, and toxicity test

All animal experiments were approved in accordance with the Institutional Animal Care and Use Committee (IACUC) of Jeil Pharmaceutical Corporation and the ethical guidelines of the Ethics Review Committee for Animal Experimentation at Handong Global University (protocol #HGU-2017–0323). All animal procedures were conducted in compliance with the guideline of the Korean Association for Laboratory Animal Science. Every effort was made to minimize the number of animals used and any suffering of the animals used in this study. Experimental animals were purchased from Koatech (Pyeongtaek, Korea). The pharmacokinetics of NPP B1 was compared with that of amphotericin B in male SD rats. The animals (weighing 300 ± 20 g) were divided into two groups of three animals each and administrated intravenously with NPP B1 1 mg/kg or amphotericin B 0.5 mg/kg. Dosing solution was prepared in DMSO/Cremophor EL/Distilled water (1/1/8, v/v) and administrated at a dose volume of 5 mg/kg. Blood samples were collected from the jugular vein and 0.05, 0.25, 0.5, 1, 3, 5, 8 and 24 hr after NPP B1 administration. Sample were centrifuged at 3,000 rpm for 10 min. The plasma concentration was quantified using LC/MS/MS. The pharmacokinetic parameters, AUC, CL, V_ss_ were calculated using noncompartmental analysis with PK Solutions 2.0 (Summit Research Services, Montrose, CO, USA).

The systemic infection animal model was established using 4-week-old male ICR mice. Five *C*. *albicans* strains, consisting of two standard (*C*. *albicans* SC5314 and KCTC7965) and three clinical (*C*. *albicans* SL23, SL28, and SL38) strains were intravenously injected into the lateral tail vein of the various mouse groups. The mice were daily monitored for signs and mortality, and then they were euthanized. Subsequently, the target pathogenic *C*. *albicans* strain and optimal concentration for systemic infection for *C*. *albicans* were determined. A clinical isolate of *C*. *albicans* (SC5314) was grown and subcultured at 35 °C on Sabouraud dextrose agar (SDA) before infection. Candidiasis was induced by injecting 5 × 10^5^ CFU/mouse via the tail vein of six 4-week-old ICR male mice. Each animal in the treatment group was administered a subcutaneous injection of reconstituted polyene macrolides 4 h after the infection. The drug efficacy was investigated by determining the survival rates of the mice.

All *in vivo* mouse toxicity studies were conducted according to Testing Guidelines for Safety Evaluation of Drugs^32^ and the Organisation for Economic Co-operation and Development (OECD) Guideline^33^. Male and female ICR mice were used 7 weeks old for the toxicity test. Groups of five mice were administrated with single and 7-day repeated intravenous doses of NPP B1 or amphotericin B (reconstituted in 10% DMSO, 10% cremophor El, and 80% saline) via the tail vein at the rate of 2 mL/min. The single doses were 1, 2.5, and 5 mg per kg body weight. For the 7-day repeated doses consisted of NPP B1 1.5 mg per kg body weight and amphotericin B 0.5 and 1.5 mg per kg body weight. Single dose toxicity measured the mortality, clinical signs, necropsy and changes on the body weight were observed during 7 days after single intravenous treatment of NPP B1 or amphotericin B. Repeated dose 7-day toxicity measured the mortality, changes on body weight, clinical signs, hematology, serum biochemistry, gross observation and organ weight of principle organs.

## Electronic supplementary material

Supplementary Information
