# Supplementary material for: Nystatin-like Pseudonocardia polyene B1, a novel disaccharide-containing antifungal heptaene antibiotic
Source: Sci Rep. 2018 Sep 11;8:13584. doi: 10.1038/s41598-018-31801-y (PMC6134108; doi:10.1038/s41598-018-31801-y)
Supplement: Supplementary file 1 — Supplementary Information [file 41598_2018_31801_MOESM1_ESM.docx]

**Nystatin-like *Pseudonocardia* polyene B1, a novel disaccharide-containing antifungal heptaene antibiotic**

Hye-Jin Kim^1^, Chi-Young Han^1^, Ji-Seon Park^2^, Sang-Hun Oh^3^, Seung-Hoon Kang^1^, Si-Sun Choi^1^, Jung-Min Kim^2^, Jin-Hwan Kwak^3^, and Eung-Soo Kim^1*^

*^1^Department of Biological Engineering, Inha University, Incheon, 22212, Korea*

*^2^Jeil Pharmaceutical Co., Ltd., Yongin-si, Gyeonggi-do, 17172, Korea*

*^3^School of Life Science, Handong Global University, Pohang, 37554, Korea*

* Corresponding author

E-mail: eungsoo@inha.ac.kr; Phone: +82-32-860-8318; Fax: +82-32-865-4046

**Supporting Information Legends**

**Figure S1.** **Development of NPP B1 producing strain through inactivation of the ER5 domain.** (A) A scheme of ER5 domain inactivation using pKC1132. (B) NPP B1 production yields from ER5 domain inactivation mutants.

**Figure S2. A comparison between NPP A1 producing strain (wild-type) and NPP B1 producing strain (ER5 domain inactivation mutant). These strains were cultured based on 3L fermentation.** (A) Dried cell weights (DCW) and production yields of these strains during cultivation. (B) The quantitative analysis of transcription levels; open circle, transcripts from the wild-type at 24hr after inoculation; closed circle, transcripts from the wild-type at 36hr after inoculation; open diamond, transcripts from the ER5 domain inactivation mutant at 24hr after inoculation; closed diamond, transcripts from the ER5 domain inactivation mutant at 36hr after inoculation; regulatory genes related to NPP A1 and NPP B1 biosynthesis, *nppRI*(light purple), *nppRII*(red), *nppRIII*(yellow), *nppRIV*(emerald green), *nppRV*(sky blue) and *nppRVI*(blue); PKS genes, *nppA*(dark blue), *nppC*(brown) and *nppI*(dark purple); house-keeping gene, *rpoD*(light green).

**Figure S3. Overexpression of pathway-specific regulatory gene (*nppRIV*).** (A) The plasmid map for overexpression of *nppRIV* gene under the control of constitutive promoter, *ermE** promoter. (B) NPP A1 and NPP B1 production yields through overexpression of *nppRIV* gene in *P. autotrophica* and ER5 domain inactivation mutant strain, individually.

**Figure S4. Identification and deletion of the global down-regulator (WblA) ortholog in NPP B1 producing strain.** (A) Multi-alignment of WblA orthologs in various *Streptomyces*. (B) NPP B1 production yields in WblA ortholog deletion mutants.

**Figure S5. *In-situ* Raman microspectroscopic screening.** (A) Raman spectra obtained from the NPP A1 and NPP B1 producing strain, *P. autotrophica* wild-type(top) and ER5 domain inactivation mutant strain(bottom). (B) Position-dependent changes of Raman spectra from the edge areas and center of ER5 domain inactivation mutant strain mycelia. (C) NPP B1 production yields from the colonies classified three group by Raman intensity; light gray, low Raman intensity; dark gray, moderate Raman intensity; black, high Raman intensity.

**Figure S6. Effect of combined cultures with (A) *C. glutamicum* (B) xenobiotics, triclosan.** (A) NPP B1 producing strain were cultured with 1-day or 2-day cultured *C. glutamicum* in YEME medium. (B) NPP B1 producing strain were cultured with various concentrations of triclosan.

**Figure S7. Prediction of secondary metabolite biosynthetic gene cluster of *P. autotrophica* originated plasmid (125kb).**

**Figure S8. The toxicities of amphotericin B and NPP B1 against two hepatocyte cell lines (Hep3B and HepG2).**

**Figure S9. Construction of mouse systemic infection model.** (A) Selection of pathogenic *C. albicans* strains. (B) MLD(Minimal Lethal Dose) test of selected *C. albicans* strain.

**Figure S1**

**
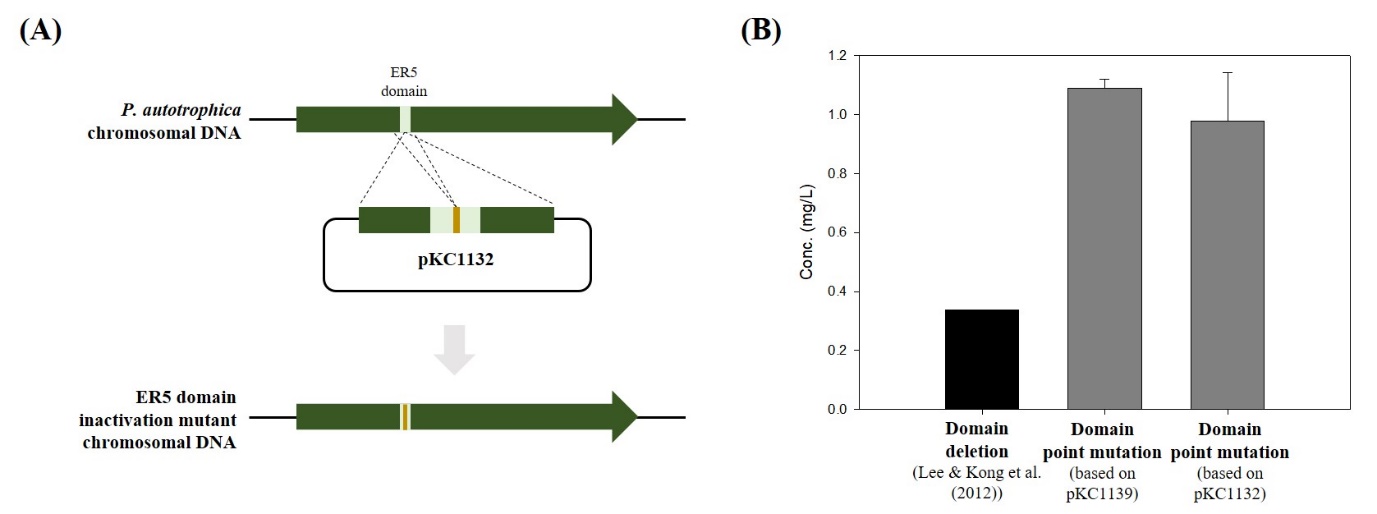
**

**Figure S2**

**
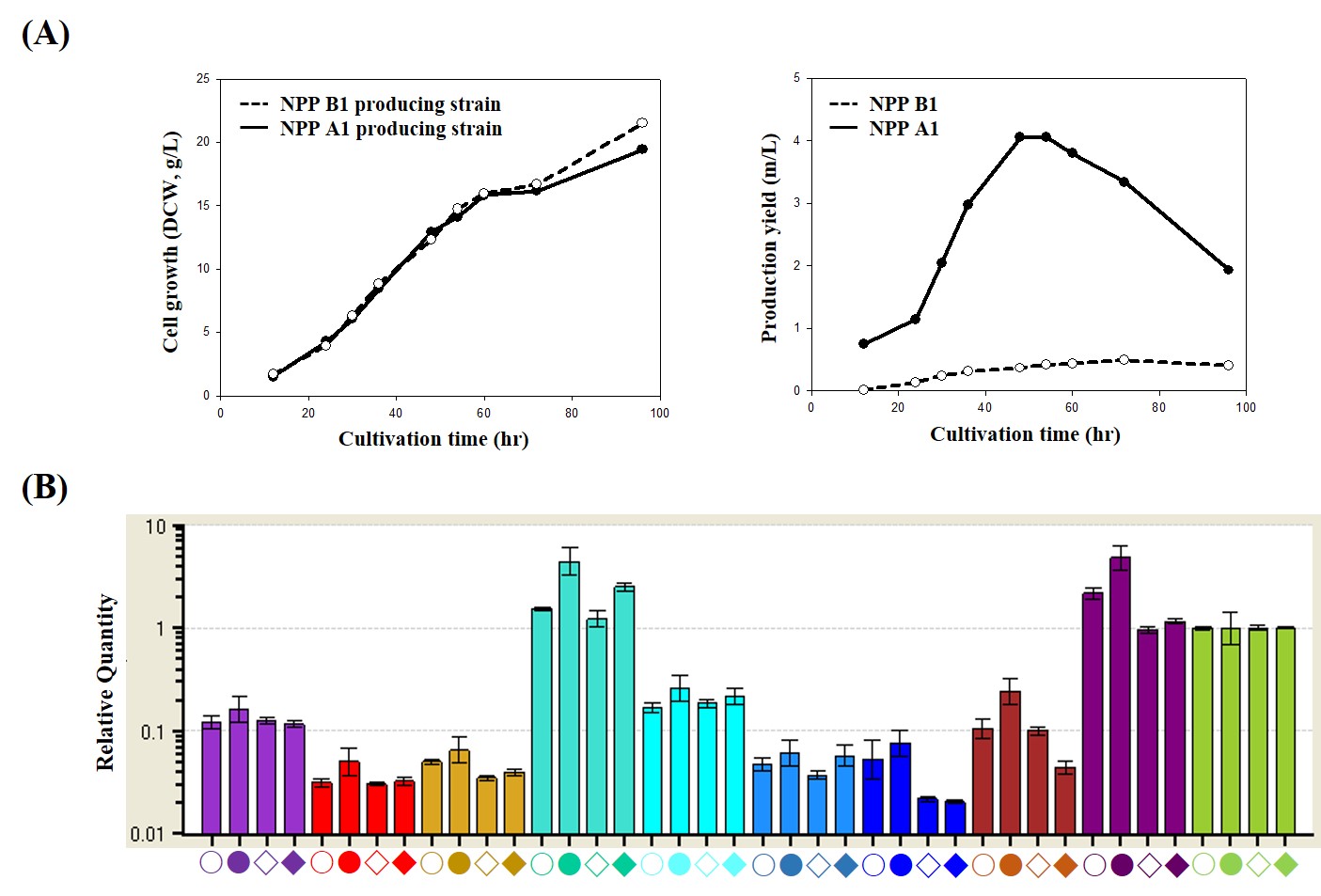
**

**Figure S3**

**
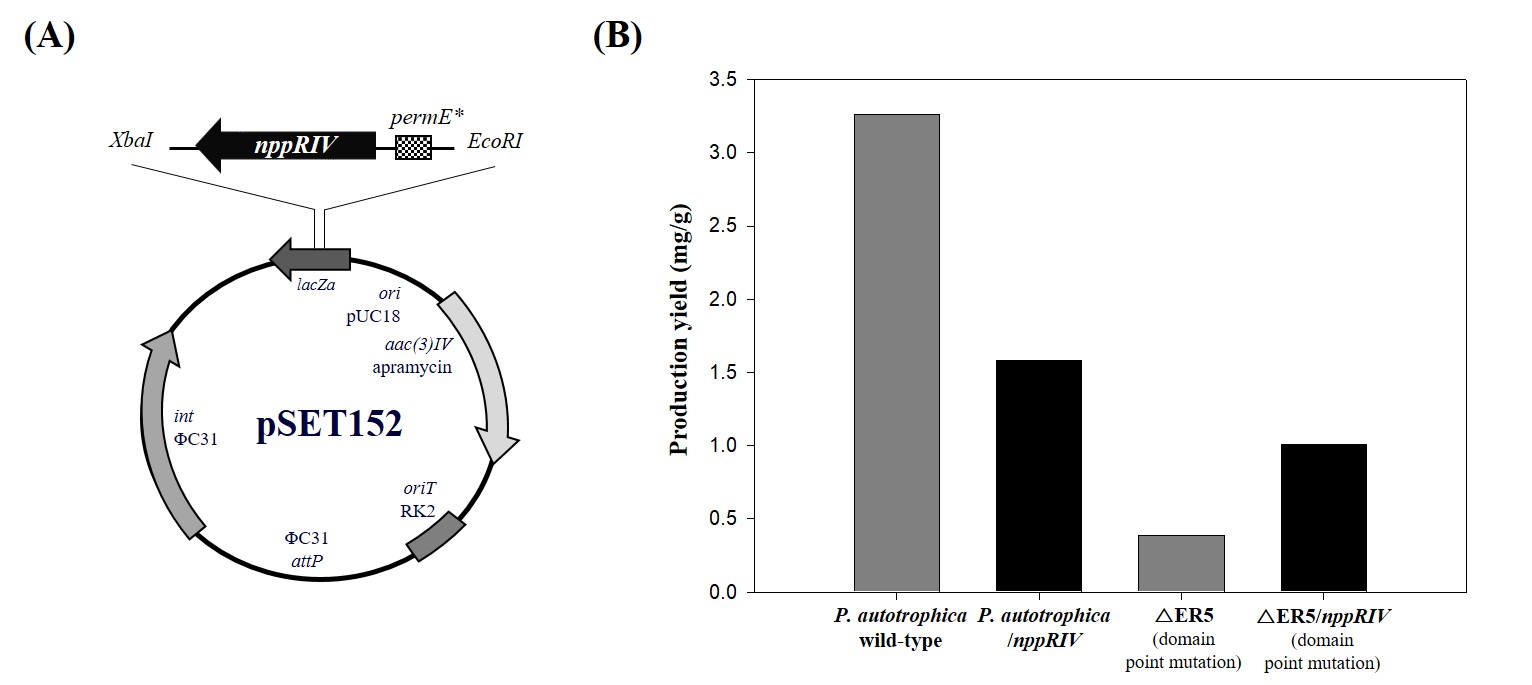
**

**Figure S4**

**
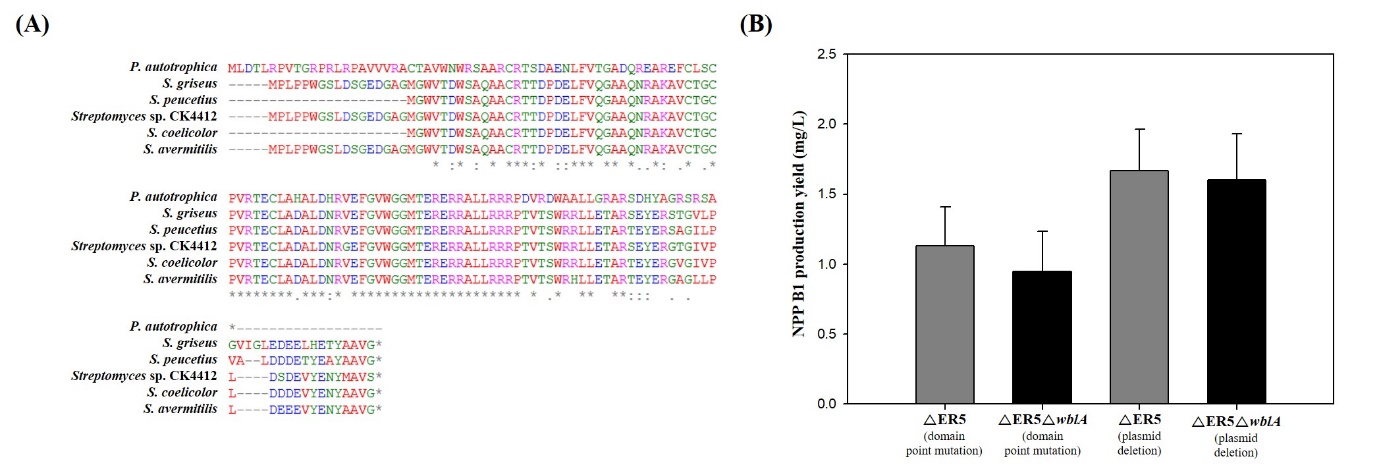
**

**Figure S5**

**
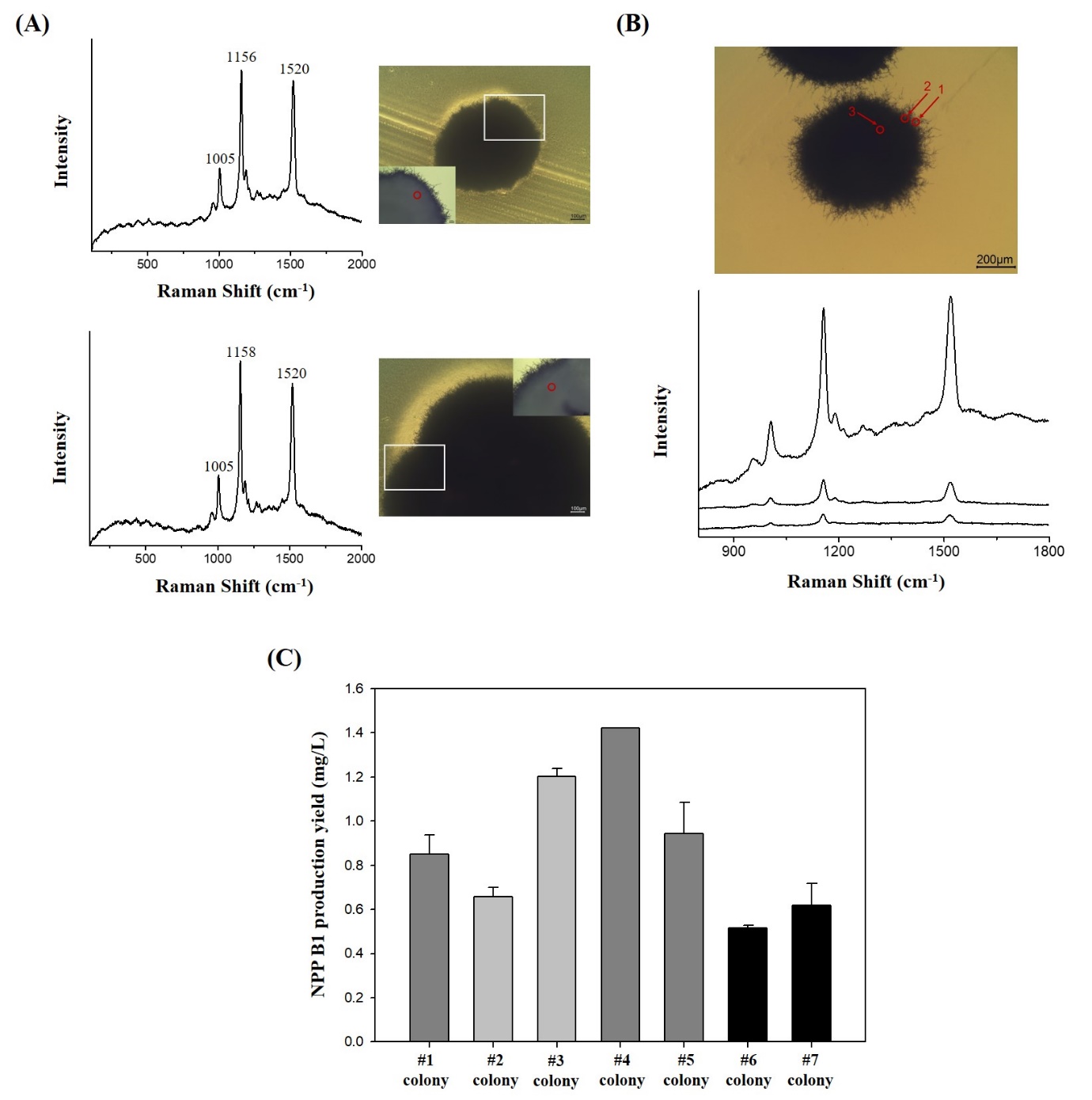
**

**Figure S6**

**
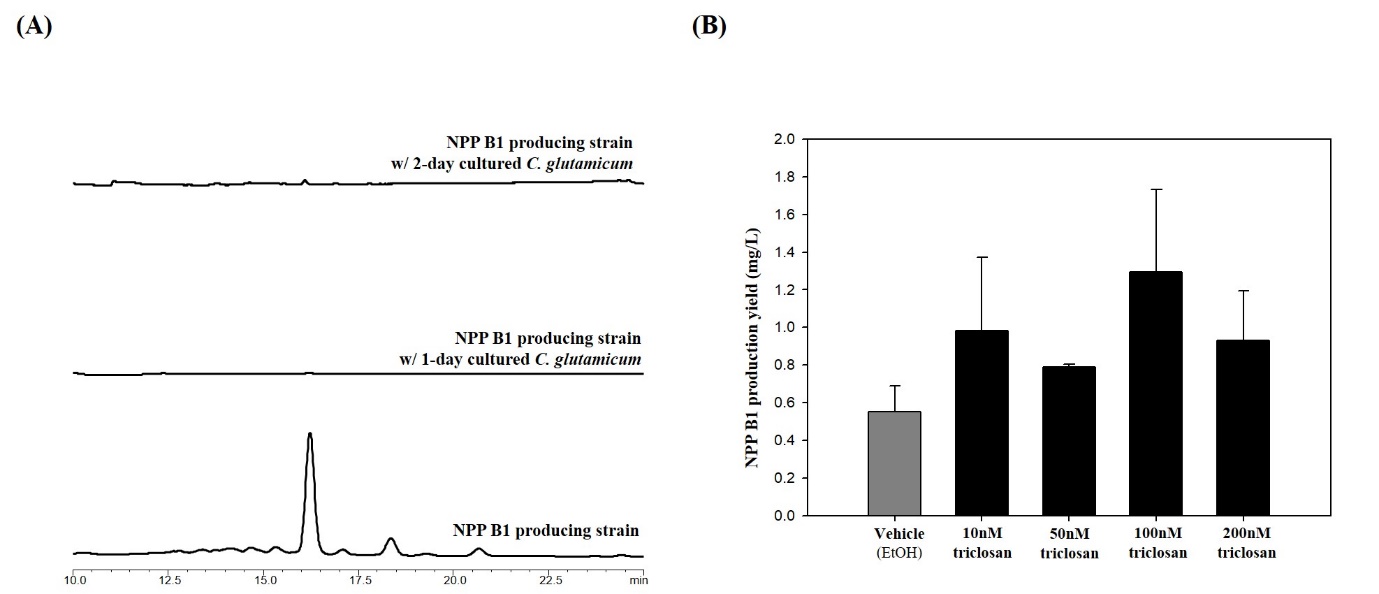
**

**Figure S7**

**
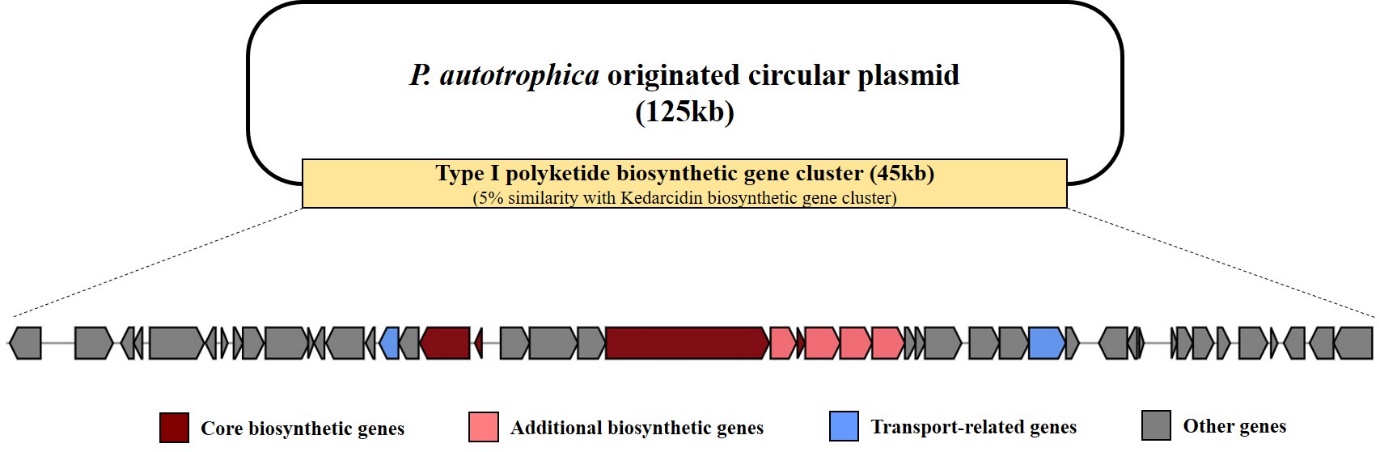
**

**Figure S8**

**
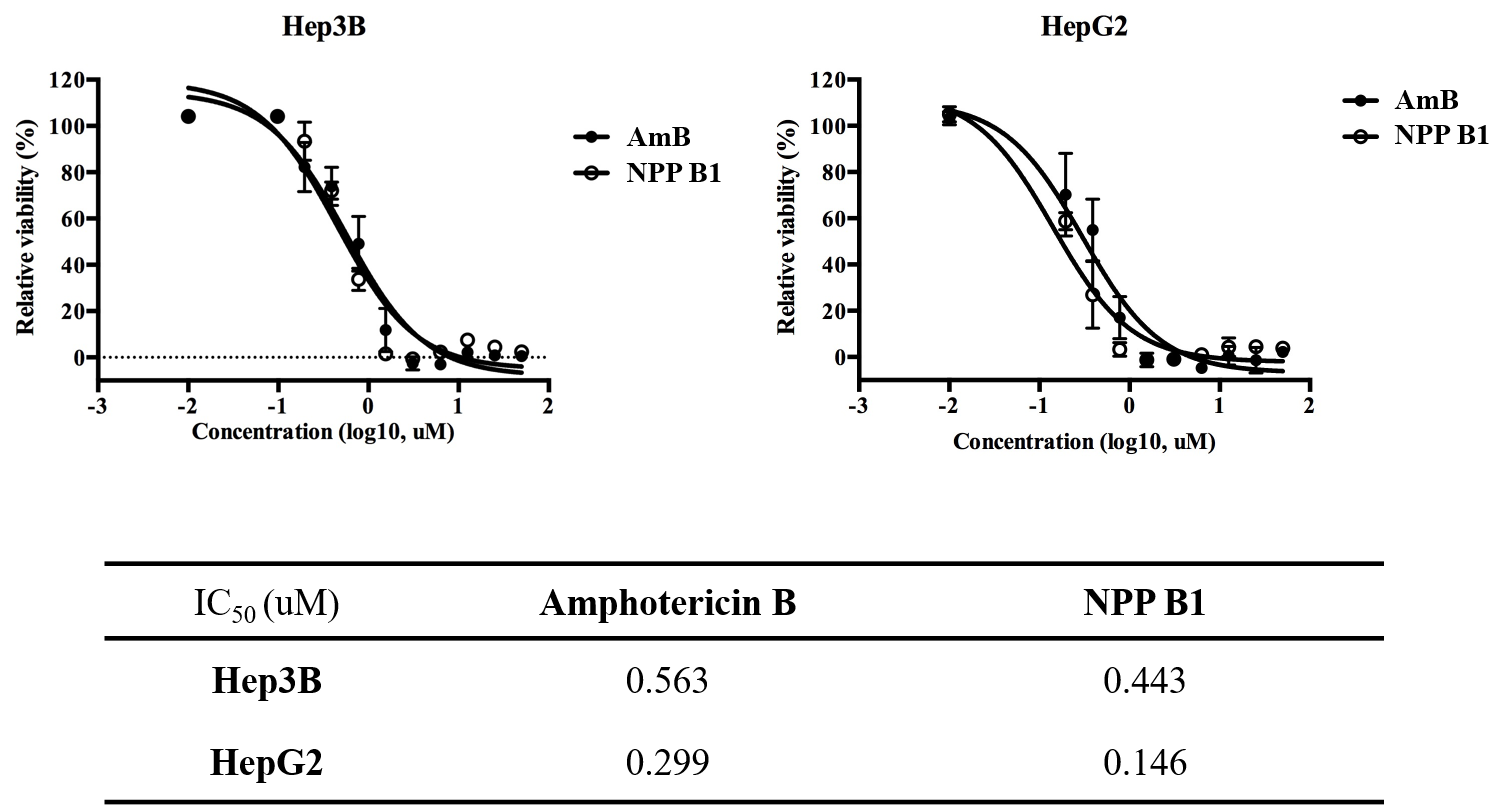
**

**Figure S9**

**
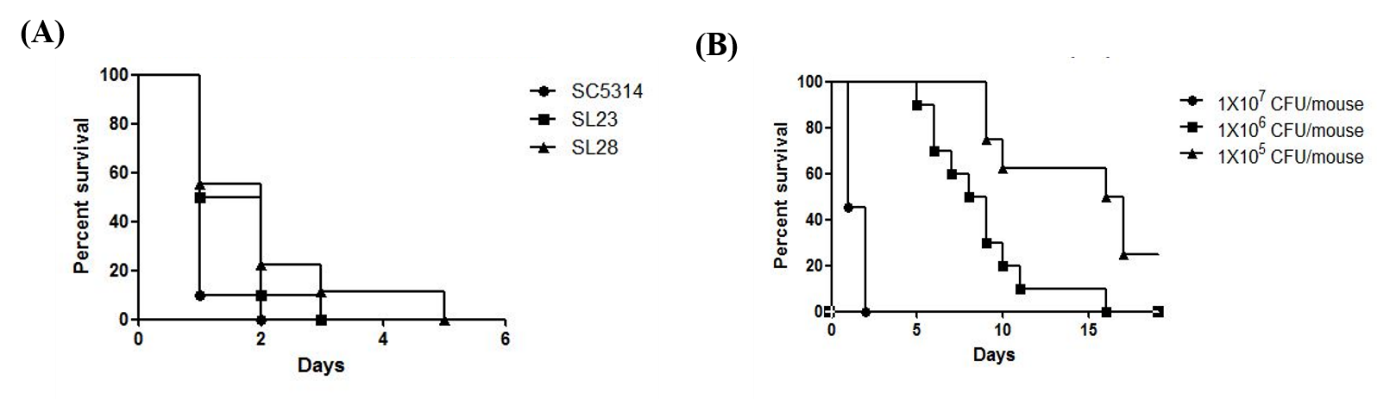
**

**Table S1. Raman intensities by NPP B1 producing colonies.**

| **Colony No.** | **Raman intensity (cm^-1^)** | **Classification** |
| --- | --- | --- |
| 1 | 33814.36 | Moderate |
| 2 | 13148.73 | Low |
| 3 | 11413.86 | Low |
| 4 | 27441.54 | Moderate |
| 5 | 25456.81 | Moderate |
| 6 | 60113.30 | High |
| 7 | 85474.81 | High |

**Table S2. Microsomal stability of NPP B1.**

|  | **% remaining** | **k** | **T_1/2_**  (min) | **CL_int_**  **_(ml/min/mg protein)_** | **Clearance class** |
| --- | --- | --- | --- | --- | --- |
| **NPP B1** | 28.2 | 0.019 | 36.5 | 19.0 | Moderate |
| **Amphotericin B** | 100.9 | NA | >80 | <8.6 | Low |
| **NPP A1** | 60.9 | NA | >80 | <8.6 | Low |
| **Nystatin A1** | 115.1 | NA | >80 | <8.6 | Low |

*Microsomal conc. : 1.0 mg/ml

**Table S3. *In-vivo* rat pharmacokinetics of NPP B1 and polyene macrolides.**

| **Parameters** | **Unit** | **Nystatin A1** | **NPP A1** | **Amphotericin B** | **NPP B1** |
| --- | --- | --- | --- | --- | --- |
| N | Number | 3 | 3 | 2 | 3 |
| Dose | mg/kg | 1 | 1 | 0.5 | 1 |
| AUC_0-t_ | ng/ml/hr | 5125.0 | 4387.5 | 415.4 | 10330.8 |
| AUC_0-inf_ | ng/ml/hr | 5304.1 | 4468.2 | 1680.9 | 13584.1 |
| CL | L/hr/kg | 0.2 | 0.2 | 0.3 | 0.1 |
| V_ss_ | L/kg | 0.2 | 0.1 | 3.5 | 1.1 |
| t_1/2_ | hr | 0.6 | 0.6 | 8.3 | 14.5 |
| MRT_inf_ | hr | 0.8 | 0.7 | 11.6 | 15.6 |

**Table S4. *In-vivo* mouse pharmacokinetics of NPP B1 and polyene macrolides.**

| **Parameters** | **Unit** | **Nystatin A1** | **NPP A1** | **Amphotericin B** | **NPP B1** |
| --- | --- | --- | --- | --- | --- |
| N | Number | 3 | 3 | 3 | 3 |
| Dose | mg/kg | 1 | 1 | 1 | 0.5 |
| AUC_0-t_ | ng/ml/hr | 7124.2 | 12422.2 | 22194.5 | 5887.9 |
| AUC_0-inf_ | ng/ml/hr | 7151.2 | 12704.2 | 29332.4 | 15067.0 |
| CL | L/hr/kg | 0.1 | 0.1 | 0.03 | 0.03 |
| V_ss_ | L/kg | 0.1 | 0.1 | 0.2 | 0.6 |
| t_1/2_ | hr | 0.6 | 1.0 | 3.4 | 12.3 |
| MRT_inf_ | hr | 0.9 | 1.1 | 5.5 | 17.0 |

**Table S5. Single intravenous treatment of NPP B1 and amphotericin B.**

| **Groups** | **Day after start of treatment** | | | | | | | | **Mortalities** |
| --- | --- | --- | --- | --- | --- | --- | --- | --- | --- |
|  | **0** | **1** | **2** | **3** | **4** | **5** | **6** | **7** |  |
| **Vehicle control** | | | | | | | | | |
| Male | 0 | 0 | 0 | 0 | 0 | 0 | 0 | 0 | 0/5 (0%) |
| Female | 0 | 0 | 0 | 0 | 0 | 0 | 0 | 0 | 0/5 (0%) |
| **Amphotericin B treated male groups** | | | | | | | | | |
| 1mg/kg | 0 | 0 | 0 | 0 | 0 | 0 | 0 | 0 | 0/5 (0%) |
| 2.5mg/kg | 0 | 0 | 2 | 0 | 0 | 0 | 0 | 0 | 2/5 (40%) |
| 5mg/kg | 5 | 0 | 0 | 0 | 0 | 0 | 0 | 0 | 5/5 (100%) |
| **Amphotericin B treated female groups** | | | | | | | | | |
| 1mg/kg | 0 | 0 | 1 | 0 | 0 | 0 | 0 | 0 | 1/5 (20%) |
| 2.5mg/kg | 0 | 1 | 3 | 0 | 0 | 0 | 1 | 0 | 5/5 (100%) |
| 5mg/kg | 4 | 0 | 1 | 0 | 0 | 0 | 0 | 0 | 5/5 (100%) |
| **NPP B1 treated male groups** | | | | | | | | | |
| 1mg/kg | 0 | 0 | 0 | 0 | 0 | 0 | 0 | 0 | 0/5 (0%) |
| 2.5mg/kg | 1 | 0 | 1 | 0 | 0 | 0 | 0 | 0 | 2/5 (40%) |
| 5mg/kg | 4 | 0 | 1 | 0 | 0 | 0 | 0 | 0 | 5/5 (100%) |
| **NPP B1 treated female groups** | | | | | | | | | |
| 1mg/kg | 0 | 0 | 0 | 0 | 0 | 0 | 0 | 0 | 0/5 (0%) |
| 2.5mg/kg | 1 | 0 | 1 | 0 | 0 | 0 | 0 | 0 | 2/5 (40%) |
| 5mg/kg | 3 | 2 | 0 | 0 | 0 | 0 | 0 | 0 | 5/5 (100%) |

**Table S6. Continuous intravenous treatment of NPP B1 and amphotericin B.**

| **Groups** | **Day after start of treatment** | | | | | | | | **Mortalities** |
| --- | --- | --- | --- | --- | --- | --- | --- | --- | --- |
|  | **0** | **1** | **2** | **3** | **4** | **5** | **6** | **7** |  |
| **Vehicle control** | | | | | | | | | |
| Male | 0 | 0 | 0 | 0 | 0 | 0 | 0 |  | 0/5 (0%) |
| Female | 0 | 0 | 0 | 0 | 0 | 0 | 0 | 0 | 0/5 (0%) |
| **Amphotericin B treated male groups** | | | | | | | | | |
| 0.5mg/kg | 0 | 0 | 0 | 0 | 0 | 0 | 0 |  | 0/5 (0%) |
| 1.5mg/kg | 0 | 0 | 0 | 0 | 0 | 0 | 1 |  | 1/5 (20%) |
| **Amphotericin B treated female groups** | | | | | | | | | |
| 0.5mg/kg | 0 | 0 | 0 | 0 | 0 | 0 | 0 | 0 | 0/5 (0%) |
| 1.5mg/kg | 0 | 0 | 0 | 0 | 0 | 0 | 0 | 0 | 0/5 (0%) |
| **NPP B1 treated male groups** | | | | | | | | | |
| 1.5mg/kg | 0 | 0 | 0 | 0 | 0 | 0 | 0 |  | 0/5 (0%) |
| **NPP B1 treated female groups** | | | | | | | | | |
| 1.5mg/kg | 0 | 0 | 0 | 0 | 0 | 0 | 0 | 0 | 0/5 (0%) |

**Table S7. Bacterial strains and plasmids used in this study.**

| Strains or plasmids | Description | Reference |
| --- | --- | --- |
| *P. autotrophica* KCTC9441 | | |
| Wild-type | NPP A1 producing strain | Kim et al. 2009 |
| △ER5 | NPP B1 producing strain, in-frame deleted ER5 domain | Lee et al. 2012 |
|  | NPP B1 producing strain, introduced mutations in NADPH binding site of ER5 domain | This study |
|  | NPP B1 producing strain, deleted the circular plasmid originated *P. autotrophica* | This study |
| *Escherichia coli* | | |
| DH5 | General cloning host |  |
| ET12567/pUZ8002 | Strain for intergeneric conjugation |  |
| Plasmids |  |  |
| pKC1139 | Bifunctional *E. coli-Streptomyces* plasmid containing temperature-sensitive origin |  |
| pKC1132 | Plasmid for homologous recombination without *Streptomyces* replicon |  |
| pWHU2653 | Template DNA for amplification of negative selection marker, CodA | Zeng et al. 2015 |
| pSET152 | Integrating vector for conjugation between *E. coli* and *Streptomyces* |  |
